# Simulated clients reveal factors that may limit contraceptive use in Kisumu, Kenya

**DOI:** 10.9745/GHSP-D-13-00075

**Published:** 2013-10-14

**Authors:** Katherine Tumlinson, Ilene S Speizer, Linda H Archer, Frieda Behets

**Affiliations:** a Gillings School of Global Public Health, University of North Carolina at Chapel Hill, Chapel Hill, NC, USA; b Carolina Population Center, University of North Carolina at Chapel Hill, Chapel Hill, NC, USA; c Jhpiego, an affiliate of Johns Hopkins University, Nairobi, Kenya

## Abstract

A better understanding of the factors influencing use of family planning has the potential to increase contraceptive prevalence and improve the ability of women and their partners to freely choose the number and spacing of their children. Investigations into factors contributing to unmet need frequently rely on data collected using household surveys or interviews with family planning clients and providers. Our research utilizes qualitative information resulting from simulated client visits to investigate programmatic barriers to contraceptive use in a sample of 19 health care facilities in Kisumu East District, a city in western Kenya. Simulated client reports indicate deficiencies in provider competence as well as tenuous relations between providers and clients. In addition, simulated client data reveal occasional absences of providers during normal facility hours of operation and requests of informal fees for services. Trainings that address specific gaps in provider medical knowledge and counseling skills as well as client-provider relations may reduce such programmatic barriers to contraceptive use. In addition, improved supervision and oversight at facilities may increase physical and financial access to services. Future research investigating provider motivations may illuminate root causes of programmatic barriers.

## Abstract (precis)

While the quality of family planning service delivery was often good, clients reported barriers including: excessively long waiting times, provider absences, informal fees, inappropriate pregnancy tests, misinformation, and provider disrespect. Improved monitoring and oversight of facility practices and examination of provider needs and motivations may increase quality of service.

## BACKGROUND

The life-saving benefits of family planning to both mother and child are well established.^1^^–^^5^ In the past 20 years alone, maternal deaths in developing countries have been reduced by 40% in response to increased access to contraceptive services.^6^ Yet, despite the success of many family planning programs in Asia and Latin America over the past 60 years, fertility rates in sub-Saharan Africa remain high.^1^ At 5.2, the total fertility rate (TFR) for sub-Saharan Africa is more than twice the global average.^7^^–^^9^ Those women who prefer to space or limit births but are not using any method of contraception are considered to have an unmet need for family planning.^10^ A better understanding of the programmatic factors influencing contraceptive use may help to address the persistent unmet need in numerous African countries.

A better understanding of the programmatic factors influencing contraceptive use may help to address persistent unmet need in many African countries.

### Quality and Access

The evaluation of family planning programs in developing countries is frequently guided by frameworks first developed in the 1980s and early 1990s.^11^^,^^12^ Quality of care, hypothesized to be a key determinant of contraceptive use, is defined by the Bruce-Jain framework and includes 6 aspects: method choice, information, client relations, provider competence, follow-up mechanisms, and integration.^12^ Access to services, sometimes referred to as availability, can refer to geographic or financial accessibility as well as the ability of potential clients to gain contact with service providers at facilities where they are seeking services^11^; access has also been found to be related to use or non-use of family planning.^13^ Access to family planning services can be inhibited by certain provider practices such as use of excessively restrictive medical criteria or provider bias against certain methods; these practices are often referred to as medical barriers to family planning^11^^,^^14^^,^^15^; addressing medical barriers may facilitate improvements in quality of care.^11^

### Measuring Progress

The quality of nationally sponsored family planning programs in Kenya was first assessed in 1989.^16^ These national programs began in Kenya in 1967 in response to high fertility rates and rapid population growth.^17^^,^^18^ The first evaluation of these programs provided evidence that government-sponsored family planning programs, long criticized for “poor performance,”^17^ were beginning to show improvement in critical areas of service quality such as method choice and client treatment; however, progress was lacking in discussion and management of contraceptive side effects, as well as wait time and inquiry into the client's reproductive goals.^16^^,^^17^^,^^19^ Only a handful of studies since 1989 have used facility-level data to measure family planning service delivery quality in Kenya at a national level; a 1995 study found improvements in discussion of side effects,^19^ and a study using data from 2004 to compare public and private facilities found no differences in the technical capacity of service providers by facility type.^20^

### The Simulated Client Approach

While most investigations of facility-level factors influencing contraceptive use rely on data collected through provider and client interviews, this article takes a less common approach by describing interactions between health care providers and *simulated* family planning clients.^21^^–^^29^ The simulated client approach provides an unobtrusive means of collecting data about service delivery and is likely to provide more accurate data than approaches using client or provider interviews or third-party observations.^26^

In the simulated client approach, a trained female data collector pretends to be a new client at a health facility and undergoes a family planning counseling session.

The data presented here are part of a larger study conducted in the Kenyan city of Kisumu, located in a region with a TFR of 5.4.^30^ The study design uses the simulated client methodology to test the validity of standard data collection instruments typically employed to measure family planning service quality and infrastructure at service delivery points. These standard instruments, collectively known as the Situation Analysis, include a facility audit, an observation guide, and questionnaires for interviewing family planning clients and service providers.^31^^,^^32^ As part of our validation study, these standard instruments were employed at the same facilities where the simulated client method was used. Analysis of validation data is ongoing. The objective of this paper is to share information provided by the simulated client method that would have gone unobserved if data collection had relied solely on the standard instruments.

## METHODS

Data for this study were collected in 19 public and private health care facilities of medium to high volume located in Kisumu East District, Kenya, in 2012. In the simulated client approach to facility-level data collection, a trained female data collector pretends to be a new family planning client at a health facility and undergoes a family planning counseling session. Following the counseling session, the simulated client records or reports her observations.

For this study, 6 simulated female clients were hired and trained. Simulated clients ranged in age from 23 to 30, with parity ranging from 0 to 3 children. All 6 clients were assigned a “preferred method” of contraception to request from the provider, which allowed investigators to examine provider practices across a range of methods. Of the 6 simulated clients, 3 were assigned a preferred method of oral contraceptive pills (OCPs), 1 was assigned a preferred method of injectables, 1 the intrauterine device (IUD), and 1 the contraceptive implant. See the simulated client profiles (Table 1) for additional information on the background characteristics of each simulated client. In addition to visits from simulated clients, all 19 selected facilities participated in a facility audit, third-party observations, and interviews with exiting family planning clients and service providers.

**Table 1.** Profiles of Simulated Clients

	**Simulated Clients**					
	**1**	**2**	**3**	**4**	**5**	**6**
**Age**	27	24	28	28	23	30
**Residence**	Non-slum	Non-slum	Slum	Slum	Slum	Slum
**Marital status**	Unmarried	Unmarried	Married	Unmarried	Married	Married
**Parity**	0	0	2	1	0	3
**Preferred method**	Pills	Injectable	IUD	Pills	Implant	Pills

Simulated clients assigned to prefer OCPs were trained to accept 1-3 packs of pills when offered. Those clients assigned to prefer injectables, the IUD, or the implant were trained to conclude their counseling session before such methods could be administered in order to avoid receiving unwanted procedures. A list of culturally appropriate and credible reasons for concluding services prior to receiving commodities was determined with input from all data collectors during the 1-week training period. Some examples of credible reasons include:

- I need to ask my husband first
- Let me go think about it
- I changed my mind, I just want the condom
- I don't have the money, let me go and come back
- I want to compare with another facility

There were an estimated 108 providers offering family planning services at the 19 participating facilities. This study was designed so that each of these providers would be visited by 1 of the 6 simulated clients; however, many facilities schedule only 1 provider to offer family planning each month or each quarter (3 months). As a result, it was not possible to collect simulated client data on all family planning providers at the 19 facilities during the study period. Multiple attempts were made to collect data on different providers by sending different simulated clients back to participating facilities; these repeat visits often resulted in multiple observations of the same provider, as seen in Table 2. Of the 52 providers reached in the study, 21 providers were visited just once. In 10 of the 19 facilities, simulated clients succeeded in visiting all family planning providers working at the facility; in another 2 facilities, clients were able to visit all but 1 of the family planning providers. In the remaining 7 participating facilities, simulated clients visited between 14% and 44% of the family planning providers. Approximately 56 family planning providers within selected facilities, or 52% of all estimated providers, were not visited by a simulated client. However, a majority of providers not visited by a mystery client were not providing family planning services or were off-duty during the study period.

**Table 2.** Distribution of Simulated Client Visits Per Provider

	**Number of providers receiving client visits**	**Total simulated client visits (# providers X # client visits)**
**Providers with 1 simulated client visit**	21	21
**Providers with 2 simulated client visits**	11	22
**Providers with 3 simulated client visits**	4	12
**Providers with 4 simulated client visits**	6	24
**Providers with 5 simulated client visits**	5	25
**Providers with 6 simulated client visits**	5	30
**Total**	52 providers	134 visits

The sample of 19 facilities was selected to include all medium (11) and high volume (8) health care facilities currently providing family planning services within Kisumu East District. The volume of facilities was determined through conversations with local NGO staff and by visiting the potential facilities to determine the number of family planning clients serviced in the preceding week, according to the official patient registration log (medium  =  <10; high  =  <25). The sample included both public (14) and private (5) facilities, and all of the selected facilities also offered maternal and child health services and/or HIV-related services in addition to family planning services. Data collection took place August 1–17 and Sept 17–28, 2012. The service providers included in the study are those who were providing services on the day a simulated client attended their facility.

Simulated clients recorded their observations soon after their counseling session with a short user-friendly and objective checklist. The checklist, informed in part by MEASURE Evaluation's Quick Investigation of Quality,^32^ was designed to capture quantitative data on aspects of family planning service delivery quality, according to the Bruce-Jain framework.^12^

In addition to these quantitative data, all 6 mystery clients had the opportunity to provide any additional information they observed while at the selected facility. This additional information, provided in both written and verbal format to the study principal investigator (and first author) in an unsolicited manner at the end of each day of data collection, provides in-depth insights for this paper; where appropriate in the paper, this information is supplemented with quantitative data from the simulated client checklist. All 6 simulated clients volunteered additional information, which was not restricted to any specific topic and was subsequently entered into a Microsoft Word document and organized into 4 emergent themes: interpersonal relations, provider competence, provider accessibility, and inappropriate charges to clients. It is important to note that the quotes provided in the results section are all drawn from this informal feedback, which was not collected in a systematic manner, and are therefore not representative of high/medium volume facilities in Kisumu.

Confidentiality was a key component of the ethics training received by the simulated clients. Each simulated client was required to sign a pledge of confidentiality upon completion of the training. Facility managers were aware of and supportive of the study. The University of North Carolina at Chapel Hill (UNC-Chapel Hill) and the Kenya Medical Research Institute (KEMRI) reviewed and approved the study protocol and informed consent process for this study.

## RESULTS

The 6 simulated clients completed a total of 134 visits with 52 providers (88% of whom were female) at the 19 participating facilities. To our knowledge, providers did not identify simulated clients during their visit, although they may have been made aware of the potential for a simulated client visit by their facility supervisor, who consented to participate in the study. In 1 instance, a simulated client reported she thought her provider became suspicious because the provider brought additional staff into the examination room to observe the consultation and questioned the client's motivation to use a contraceptive method. Outside of this 1 event, all simulated clients reported that they felt confident the observed providers did not identify their true purpose.

### Client-Provider Interactions

In 5 out of the 134 simulated client visits, the client volunteered unsolicited feedback characterizing their provider as “friendly,” “respectful,” or “nice.” These 5 voluntary reports of positive client interaction reference 4 providers (1 provider received 2 positive reports) working at 2 public and 2 private facilities. Clients volunteering positive reports had expressed a preference to their provider to use either pills or implants. One client provided an account of a provider who did a good job discussing the different family planning options. Another client reported that her provider was very encouraging of the client's desire to begin a contraceptive method. The following demonstrates a provider taking steps to ensure client access to a method not currently available at the facility:

Despite the fact that the method I wanted was not available in the facility, the provider managed to tell me more about the method I had chosen and she even made a call to the family planning team which was going around in various facilities to provide family planning services which were not available in those facilities.

However, not all accounts of interactions with providers were positive. According to quantitative checklist data, providers failed to greet simulated clients in a respectful or friendly manner at 18% of visits; these 24 visits were spread across 13 different facilities, 2 of which are private, and 17 different providers. Of the 17 providers with a reportedly unfriendly manner, 16 were visited by more than 1 simulated client. Of the 16 providers with multiple visits, 4 received more than 1 negative report; in only 1 case did all simulated clients report independently that the provider was lacking in respect. Half of the negative reports came from clients assigned to prefer OCPs; this is not surprising given that half of the 6 simulated clients had this assignment. Unfriendly behavior was rarely reported by the implant client (8% of all negative reports) or the IUD client (also 8% of all negative reports). Of the negative reports, 38% came from the simulated client assigned to prefer injectable contraception; it is possible this is more a reflection of this client's age (24 years) than her preferred method.

According to the quantitative checklist tool, 1 in 5 providers displayed reportedly negative attitudes towards clients.

All 6 simulated clients voluntarily mentioned rude or disrespectful treatment at some point by 1 or more service providers visited during the study. According to informal feedback, in 2 public facilities, a provider reportedly stated “family planning is not an emergency” in an effort to explain long wait times or to appease clients who could not be seen on the same day that they arrived. As 1 client reported:

The provider was so rude… arrogant. Women were really complaining. The provider yelled at the clients and told them no one can challenge her on family planning. If she wanted to, she could tell everyone to just go home and come back another day. She said, ‘I'm tired of injecting your buttocks every day.'

In addition to the providers displaying reportedly negative attitudes towards clients (according to the quantitative checklist tool), with 1 provider going so far as to engage in such behavior as shouting at clients (according to informal feedback), 1 additional provider harbored unfounded suspicions that affected client access to desired methods. In this case, a simulated client seeking injectable contraception was strongly accused of coming to the facility knowing that she was pregnant, in the hopes that receiving an injection would induce an abortion. Due to this suspicion on the part of this provider at a public facility, the simulated client was not offered any family planning method.

### Technical Competence of Service Providers

According to a combination of quantitative checklist data and informal feedback, in 10% of all simulated client visits (13 visits with 10 different providers at 8 public facilities), the service provider refused to offer the client their preferred method of contraception unless the client was able to provide physical evidence of current menstruation or was willing to take a pregnancy test (at an additional cost of 100 to 150 Kenyan shillings, equivalent to US$1.18–$1.76). In the remaining 90% of simulated client visits, all clients were offered their preferred method or were referred to a facility where their method could be obtained.

Of the 13 instances where unnecessary menstrual requirements were imposed, 9 occurred with clients requesting OCPs, 3 during requests for injectable contraception, and in 1 instance with a client requesting the implant. In explaining this medical barrier, 1 mystery client reported:

The provider advised me to go back (to the clinic) when on menses or to do a pregnancy test so as to prove there was no pregnancy.

In no instance did any of these 10 providers attempt to rule out pregnancy by another means, such as inquiring about unprotected intercourse since the client's last menstrual period. Clients unable to meet these requirements were instructed to return at their next menses or when they had funds for a pregnancy test. In most cases, clients who were turned away were not offered an alternative method, such as condoms, for use in the meantime. Interestingly, among those providers imposing menstrual requirements and with multiple simulated client visits, some did not impose these requirements for all hormonal types or all simulated clients; for example, 2 providers imposed menstrual requirements for OCPs but no other hormonal method, while another imposed requirements only for injectables clients. Two of the providers refused to offer OCPs to some, but not all, of the simulated clients requesting this method without proof of menstruation or pregnancy test.

Simulated client reports indicate deficiencies in provider competence as well as tenuous relations between providers and clients.

In addition to medically unnecessary menstrual requirements,^15^^,^^33^ several providers reportedly dispersed misinformation to clients. For example, 1 simulated client volunteered feedback that she was sometimes discouraged from using injectable contraception due to concerns about excessive delays in the time it takes the average client to return to fertility; more than 1 provider stated average return to fertility for a client discontinuing injectable contraception is 2 years or greater. In refusing to offer injectable contraception to a simulated client, 1 provider at a public facility stated “the injection can't be given to someone who has not had kids.” In another instance, a client visiting a private facility was provided misinformation by her provider about the IUD:

My provider told me … payment depends on the type (of IUD), for example, one for 5 years costs 1,000 KSh (equivalent to US$11.76), one for 10 years costs 2,000 KSh (US$23.53), one for 15 years costs 3,000 KSh (US$35.29).”

These different versions of the copper-bearing IUD do not exist.^34^

Simulated clients also volunteered information suggesting that at least 3 of the 44 providers visited by a mystery client at a public facility may not have been trained to deliver family planning services. For example, in 1 public facility, all 6 simulated clients were offered family planning services by a person volunteering as a mentor for HIV patients. At another public facility, staff members performing patient registration or lab work also provide family planning counseling when the facility is short-staffed. It was unclear whether these personnel had adequate training in provision of family planning methods to step into this role.

### Provider Accessibility

Simulated clients frequently mentioned excessively long wait times, often due to large numbers of clients and few providers, which resulted in their inability to make contact with the targeted provider during the first attempt. For example, 2 simulated clients arrived on the same day at the same public facility shortly before 9 am, waited until 4 pm without receiving services, and were then asked to return another day. Another client arrived at a different facility at 11 am and waited until closing without receiving services; she was also told to come back another day. In total, 4 simulated clients were turned away at the end of the day without receiving services after waiting most of the day; this occurred at 3 different facilities, 1 of which was private.

Of those visits for which they were not turned away at the end of the day, simulated clients waited an average of 3 hours between arrival and departure at the facility (according to the checklist instrument), and, in 19% of visits, simulated clients waited 5 or more hours at the facility. Furthermore, those seen after an acceptable amount of wait time sometimes felt the provider was unable to offer the necessary time and attention. As 1 client reported, “The provider was in a hurry. She wanted to go for lunch and just counseled me in the hallway.”

Data reveal occasional absences of providers during normal facility hours and requests of informal fees for services.

In other cases, simulated clients mentioned that care was delayed because providers arrived late to the facility (some arriving as late as 12 pm despite official opening times of 8 am in all 19 facilities), or the facility opened late, or the providers did not return to the facility after the lunch break. This type of delayed care occurred on 7 occasions, at 7 different facilities, 2 of which were private. In cases where the provider did not return after lunch, the clients waited until closing time without ever receiving services. In 1 case, a client arrived at 2 pm on a Friday and found the provider promptly, but the provider informed the client that she was tired and asked her to come back on Monday. The provider did not offer the client any contraceptive method, such as condoms, for protection over the weekend. The official closing time on Fridays at this facility is 5 pm.

Provider accessibility was also sometimes compromised by competing duties; in a facility where no other provider was on hand that day, a simulated client reported, “The provider did not complete the service because she received a phone call telling her to go somewhere.” The client had to leave the facility without completing the family planning counseling session and without receiving any method of contraception.

### Inappropriate Charges to Clients

In 3 out of every 4 simulated client visits where the client received 1 or more packs of OCPs (a total of 57 visits), the client was charged a fee greater than the price reported by the facility manager. Often the client was charged 50 Kenyan shillings (approximately US$0.59) in a facility where the manager indicated pills are provided for free, including patient registration. In some cases (12 visits), other simulated clients attending the same facility but seeing a different provider were charged a different price or were not charged at all, indicating inconsistencies in fee collection within facilities. On 2 occasions, service providers refused to provide clients with receipts and were observed putting the fee directly into their pockets while still in the closed door counseling or examination room. Of the 14 facilities engaging in informal fee collection, 3 are private facilities. Because simulated clients were unable to accept invasive or unwanted procedures (such as an injection, IUD, or implant) for ethical reasons, we were unable to ascertain whether inappropriate fees are charged for methods other than OCPs.

## DISCUSSION

These data, resulting from 134 simulated client visits with 52 providers in 19 public and private facilities, provide information on family planning service provision in Kisumu East District as it would occur naturally, in the absence of a data collection team. Simulated clients reported rude or disrespectful treatment by a number of providers, including shouting and unfounded accusations, and clients reported receiving services by 3 possibly untrained staff. Medical barriers were also observed, including unnecessary menstrual requirements and misinformation resulting from provider bias against injectable contraception for nulliparous women. Simulated clients sometimes waited at a facility for an entire day without receiving services and were often charged fees for services greater than the price reported in the corresponding facility audit.

Much of the information shared in this paper is similar to other studies using the simulated client method, which found frequent implementation of menstrual requirements^21^ and disrespectful treatment by providers.^22^^,^^27^^,^^28^ Some informal information volunteered by the simulated clients, such as the garnering of informal fees and waiting most of the day at a facility without receiving any services, has not been seen in previous results from simulated client studies.

The implications of this study are that service quality deficiencies, medical barriers, and access issues related to provider availability and inappropriate client charges may limit clients' ability to obtain the family planning services for which they come to health facilities. In addition, women who are treated with disrespect and given misinformation may spread the word to others who might subsequently decide not to visit those facilities.

Regarding consistency of findings, there was no overall discernible pattern in the aspects of poor delivery across the participating facilities or providers. The facilities where providers were unfriendly or rude were not always the facilities where menstrual requirements were imposed or providers were absent. Notably, a facility in which a provider was twice characterized as encouraging and friendly by simulated clients was also 1 of the 5 facilities in which none of the providers engaged in collection of inappropriate client fees.

In considering the rights of clients to have access to high-quality family planning services, free of unnecessary medical barriers, it is important to first think carefully about the rights and needs of family planning service providers. The ability to provide services in a technically competent manner depends on adequate training, updated technical information, necessary equipment and supplies, and appropriate guidance.^35^ Respectful treatment of clients and consistent accessibility can be better ensured by providers with a manageable workload, timely and adequate pay, and respectful workplace practices.^35^ Efforts to better understand the perspective, needs, and motivations of the service providers are essential for identifying root causes of poor service quality and may help to address quality of care deficiencies and medical barriers identified in this paper. As other researchers have pointed out, findings from quality of care studies are not meant to “attack” providers, who are often “doing what they think best for their clients”^14^; therefore, studies designed to capture provider perspectives should be a priority in client-centered programs.

### Programmatic Implications

#### Additional Training for Facility Staff

The disrespectful manner reported in the checklist appears widespread, while the shouting and unfounded suspicions were less commonly mentioned by the simulated clients. However, both the quantitative and qualitative information from simulated clients regarding their interactions with facility staff suggest the need for additional training in counseling skills to improve interactions with clients. Even those providers with an impressive knowledge base regarding a variety of available family planning methods may fail to meet the contraceptive needs of their clients if they are engaging with clients in a rude or dismissive manner.

Quantitative and qualitative information suggests the need for additional facility staff training in counseling skills to improve interactions with clients.

#### Use Tools Such as the Pregnancy Checklist

The presence of medical barriers may also impede client access to family planning methods. Requiring evidence of menstruation or requiring a pregnancy test before providing family planning is a common barrier. Those women who cannot afford a pregnancy test and must wait until their next menses to receive a method are at risk of an unintended pregnancy in the interim. According to the World Health Organization, hormonal methods pose no medical danger to women or their pregnancy if accidentally used while pregnant (with the exception of the intrauterine device; this method should not be inserted during pregnancy).^36^ Those providers wishing to be reasonably certain their client is not pregnant can use a simple job aid developed by FHI 360: the Pregnancy Checklist.^37^ Training providers on consistent and proper use of the pregnancy checklist in facilities where pregnancy tests are not freely available has the potential to increase contraceptive uptake.^38^

#### Use of Accurate Information by Trained Personnel

The provision of misinformation to clients resulting from provider bias is another medical barrier to accessing family planning methods revealed in this study. The average delay in return to fertility for women using Depo-Provera is 9 months after their last injection.^39^^,^^40^ Providers who mistakenly believe that average return to fertility for injectable contraception is 2 or more years may deny or discourage use of this highly effective method in younger, childless, or low-parity women. This study also revealed the possibility of unqualified staff members providing family planning counseling on occasions when the volume of clients could not be met by available providers. Such practices could potentially result in harm to the client if these staff members have not been trained in family planning provision.

#### Better Supervision

It is important to ensure that providers arrive on time and are committed to providing services during the facility's posted hours of operation. It may be beneficial to reduce the number of legitimately competing priorities that pull providers away from their facilities during peak hours of service delivery. In addition, creating a more rigorous system of management and supervision may help to ensure that providers are not frequently away on personal business during working hours.

Improved supervision and oversight at facilities may increase physical and financial access to services.

#### Transparent Fees

Lastly, this study reveals an informal fee structure that suggests possibly corrupt behavior on the part of some providers, which could create financial barriers to contraceptive services, particularly among low-income clients. Forty percent of Kenyans currently live on less than US$2 per day.^41^ Therefore, even a small informal fee of US$0.59 may constitute a significant portion of income for the average family planning client. The informal fee structure revealed in this study appears to be fairly widespread for OCPs among the facilities included in the study; implementing mechanisms such as receipt books or publicly displayed prices to help discourage corruption may lead to increased contraceptive use.

### Limitations of This Study

The simulated client method allows the researcher to collect information on actual practice that would be difficult to obtain through other means. However, this method is not without limitations. First, there is the possibility of poor recall or subjective interpretation on the part of the simulated client. To address this concern, the 6 simulated clients who collected the data for this study participated in extensive training and pilot testing of their data collection instruments. All records and reports from each visit to a participating facility were submitted to the study's principal investigator on the same day as the visit, and opportunities for clarification or elucidation were provided as needed.

A second challenge with this methodology is the recruitment of simulated clients who realistically represent different sections of the population, including residents of areas with slum-like conditions. All 6 simulated clients were residents of Kisumu East District and resided in the catchment area of 1 or more of the facilities included in the study.

An additional limitation of the simulated client method is the 1-sided perspective of this approach to data collection. While the methodology allows for unobtrusive observation of provider performance, it does not consider the perspective of the provider or deficiencies in training, infrastructure, supervision or other general areas of support that may be lacking in the provider's work environment.^35^

Lastly, it is important to note that, given the design of the study, it is not possible to generalize these findings to all health care providers or facilities in Kisumu East District. However, many of the practices reported above are happening in 1 or more facilities and therefore warrant examination and further attention.

## CONCLUSION

The simulated client method allows researchers to collect information on service delivery practices as they occur naturally, in the absence of data collectors and research staff, and therefore can provide critical insights into aspects of care that may limit contraceptive use. Much of the quantitative and qualitative information supplied by the simulated clients in this study would have been difficult or impossible to collect via facility audits, third-party observations, or interviews with clients and staff. The results point to important issues around quality of care, medical barriers, and provider and financial access that may be impeding use of family planning services among potential clients. A larger and more systematic simulated client study would reveal whether some of the practices identified in this paper are widespread or isolated among a few providers or facilities. Increased training and heightened supervision of providers is one possible solution to these issues, as presented above in Programmatic Implications. However, a better understanding of provider needs and motivations will also be key to understanding the root causes of barriers to contraceptive use. Addressing these barriers not only has the potential to reduce maternal and infant mortality but also is an important step in safeguarding women's reproductive rights.

Future research investigating provider motivations may illuminate root causes of programmatic barriers.
